# The Peripheral Arterial disease study (PERART/ARTPER): prevalence and risk factors in the general population

**DOI:** 10.1186/1471-2458-10-38

**Published:** 2010-01-27

**Authors:** María Teresa Alzamora, Rosa Forés, José Miguel Baena-Díez, Guillem Pera, Pere Toran, Marta Sorribes, Marisa Vicheto, María Dolores Reina, Amparo Sancho, Carlos Albaladejo, Judith Llussà, the PERART/ARTPER study group

**Affiliations:** 1 Primary Healthcare Centre Riu Nord-Riu Sud, Institut Català de la Salut, Santa Coloma de Gramenet, Spain; 2 Department of Medicine, Universitat Autònoma de Barcelona, Barcelona Spain; 3 Research Unit Metropolitana Nord, ICS-IDIAP Jordi Gol. Mataró, Spain; 4 Primary Healthcare Centre La Marina, Institut Català de la Salut, Barcelona, Spain; 5 Primary Healthcare Centre Numància, Institut Català de la Salut, Barcelona, Spain; 6 Primary Healthcare Centre Santa Coloma de Gramenet, Institut Català de la Salut, Santa Coloma de Gramenet, Spain; 7 Primary Healthcare Centre Can Mariné, Institut Català de la Salut, Santa Coloma de Gramenet, Spain; 8 Primary Healthcare Centre Llefià, Institut Català de la Salut Badalona, Badalona, Spain; 9 Primary Healthcare Centre Sant Roc, Institut Català de la Salut, Badalona, Spain

## Abstract

**Background:**

The early diagnosis of atherosclerotic disease is essential for developing preventive strategies in populations at high risk and acting when the disease is still asymptomatic. A low ankle-arm index is a good marker of vascular events and may be diminished without presenting symptomatology (silent peripheral arterial disease). The aim of the study is to know the prevalence and associated risk factors of peripheral arterial disease in the general population.

**Methods:**

We performed a cross-sectional, multicentre, population-based study in 3786 individuals >49 years, randomly selected in 28 primary care centres in Barcelona (Spain). Peripheral arterial disease was evaluated using the ankle-arm index. Values < 0.9 were considered as peripheral arterial disease.

**Results:**

The prevalence (95% confidence interval) of peripheral arterial disease was 7.6% (6.7-8.4), (males 10.2% (9.2-11.2), females 5.3% (4.6-6.0); *p *< 0.001).

Multivariate analysis showed the following risk factors: male sex [odds ratio (OR) 1.62; 95% confidence interval 1.01-2.59]; age OR 2.00 per 10 years (1.64-2.44); inability to perform physical activity [OR 1.77 (1.17-2.68) for mild limitation to OR 7.08 (2.61-19.16) for breathless performing any activity]; smoking [OR 2.19 (1.34-3.58) for former smokers and OR 3.83 (2.23-6.58) for current smokers]; hypertension OR 1.85 (1.29-2.65); diabetes OR 2.01 (1.42-2.83); previous cardiovascular disease OR 2.19 (1.52-3.15); hypercholesterolemia OR 1.55 (1.11-2.18); hypertriglyceridemia OR 1.55 (1.10-2.19). Body mass index ≥25 Kg/m^2 ^OR 0.57 (0.38-0.87) and walking >7 hours/week OR 0.67 (0.49-0.94) were found as protector factors.

**Conclusions:**

The prevalence of peripheral arterial disease is low, higher in males and increases with age in both sexes. In addition to previously described risk factors we found a protector effect in physical exercise and overweight.

## Background

The prevention and early diagnosis of atherosclerotic disease is one of the essential objectives in the field of cardiovascular disease since it is the main cause of mortality in developed countries. In the European Union these diseases represent approximately 40% of the deaths in both men and women [1]. Atherosclerosis is currently considered a chronic, progressive systemic disease of multifactorial aetiology [2] including arterial hypertension, hypercholesterolemia, diabetes mellitus and smoking as modifiable risk factors and age and sex as non modifiable factors [3]. These factors have been integrated in prediction tables based on regression models with the aim of detecting the population with a high risk of cardiovascular events [4]. However, the sensitivity and positive predictive value of these tables is low, and thus, most cardiovascular disease are produced in subjects without a high risk [4]. It is therefore important to develop markers of silent atherosclerotic disease which will help to better identify subjects at high risk of developing peripheral arterial disease in order to implement preventive measures. In addition, atherosclerotic disease remains clinically silent during most of the evolutionary process until phenomena of complications of atheroma plaques suddenly appear and lead to ischaemic vascular events. Therefore, from a primary prevention point of view, it is very important to develop strategies which allow identification of patients with atherosclerosis in subclinical stages, with primary care being the optimum setting for accessibility.

Peripheral arterial disease is a common manifestation of atherosclerosis and is characterized by increasing incidence of morbi-mortality [5]. Peripheral arterial disease is a powerful predictor of cardio and cerebrovascular events and is associated with an increase in mortality of up to 30% at 5 years and 50% at 10 years [5-7] thereby making early detection of this disease fundamental.

The prevalence of peripheral arterial disease varies greatly depending on the population studied, on the definition of peripheral arterial disease used (symptomatic or not), the diagnostic method, age, sex and the presence of other risk factors. Most studies have been carried out in populations at high risk, with few having been performed in countries of low risk [8-16]. The prevalence of peripheral arterial disease in countries with low cardiovascular risk is uncertain.

Underdiagnosis of peripheral arterial disease is very high since a very large proportion of these patients is asymptomatic [17]. The ankle-arm index is the most effective tool used to screen for peripheral arterial disease [18,19]. Compared with angiography, an ankle arm index <0.9 presents a sensitivity of 95% and a specificity of 99% for the detection of stenosis ≥50% [20], with the reliability of this test being good when performed by trained personnel [18-21].

The wide range of prevalence reported in the literature and the low number of studies in countries of low risk such as Spain led to the design of this study, the aim of which was to know the prevalence of peripheral arterial disease using the calculation of the ankle arm index in a general population over the age of 49 years and to determine the risk factors associated with a pathologic ankle arm index.

## Methods

A detailed description of the methodology of the study has been published elsewhere [22].

Briefly, this was a cross-sectional, multicentre, descriptive, population-based study aim at determining the prevalence of symptomatic and asymptomatic peripheral arterial disease and related factors in a general population ascribed in primary care. Individuals were randomly selected (simple random sampling) from a database, which is more exhaustive and updated than the census, containing the population ascribed in the centers participating in the study. The randomly selected individuals were invited to participate in the study by phone. If the individuals were not found, up to 5 calls were made at different hours and days of the week to contact them. After agreement to participate subjects were given an appointment to perform an interview, blood sample extraction and anthropometric measurements, including ankle-arm index.

A total of 28 primary healthcare centers within the metropolitan area of the city of Barcelona and the county of Barcelonès Nord-Maresme, including urban and semi-rural centers, participated in the study. Finally, from September 2006 to June 2008, a total of 3786 patients over the age of 49 years were included in the study.

### Data collection

The ankle arm index examination of the subjects was carried out in the participating centres by two healthcare professionals trained in the technique, under standardized conditions. A standardized Doppler Ultrasonic device was used (Mini-Dopplex D 900-P, Huntleigh Healthcare, 8 MHz). Ankle-arm index was performed in the two paramaleolar arteries of both lower extremities. For each leg the ankle-arm index was the ratio of the higher of the two systolic pressures (tibial posterior and anterior artery) and arm systolic pressure using the systolic pressure of the highest arm. If the ankle-arm index was < 0.9 the technique was performed by the other professional. In cases in which the second professional found an ankle arm index ≥ 0.9 the first repeated the test and the latter value was considered as the final result.

The following variables were collected: demographic and lifestyle, including smoking and physical activity with a validated questionnaire, self-reported and clinical history of previous cardiovascular disease (acute myocardial infarction, angina, stroke and transient ischaemic attack), intermittent claudication, hypercholesterolemia, diabetes mellitus, arterial hypertension; anthropometric variables (height, weight, and waist circumference), blood analysis (total cholesterol, HDL-cholesterol, triglycerides, and glycaemia); cardiovascular risk using the Framingham-Wilson equations, Framingham calibrated by the REGICOR and SCORE groups; metabolic syndrome (NCEP criteria) [23]; and the Edinburgh vascular questionnaire [8,15].

### Statistical analysis

The prevalence of peripheral arterial disease was computed in the whole sample. However, patients with Mönckeberg sclerosis, indicated as an ankle arm index >1.4 (arterial calcification) were excluded from further analyses involving potential associated risk factors with peripheral arterial disease. Comparison of categorical variables was performed using chi squared tests and for continuous variables the t-test was used. In addition, non parametric tests were used when distribution requirements of the variables analyzed were not fulfilled. Logistic regression models (LRM) were adjusted using peripheral arterial disease as the dependent variable. The individual effect, measured with the odds ratios [OR, (95% confidence interval)] of the potential risk factors, was studied in age- and sex-adjusted LRM. Multivariate LRM were thereafter constructed to assess the independent effects of each variable adjusted for the other in the model. Best models were selected taking into account the correlation between variables and the Akaike Information Criteria (AIC) [24]. Only variables with p < 0.05 remained in the model. Significance was defined with a p value less than 0.05. All tests were bilateral. Analysis was performed using Stata version 10 (StataCorp, College Station, TX, USA, 2007).

### Ethics

This study was approved by the local Ethics Committee (IDIAP Jordi Gol Foundation of Investigation in Primary Care and Instituto de Salud Carlos III). Informed written consent was obtained from all the participants. Likewise, the recommendations of the World Medical Association Declaration of Helsinki were follow.

### Ethical considerations

The authors of this manuscript have certified that they comply with the Principles of Ethical Publishing in BMC Public Health.

## Results

**Figure 1:**
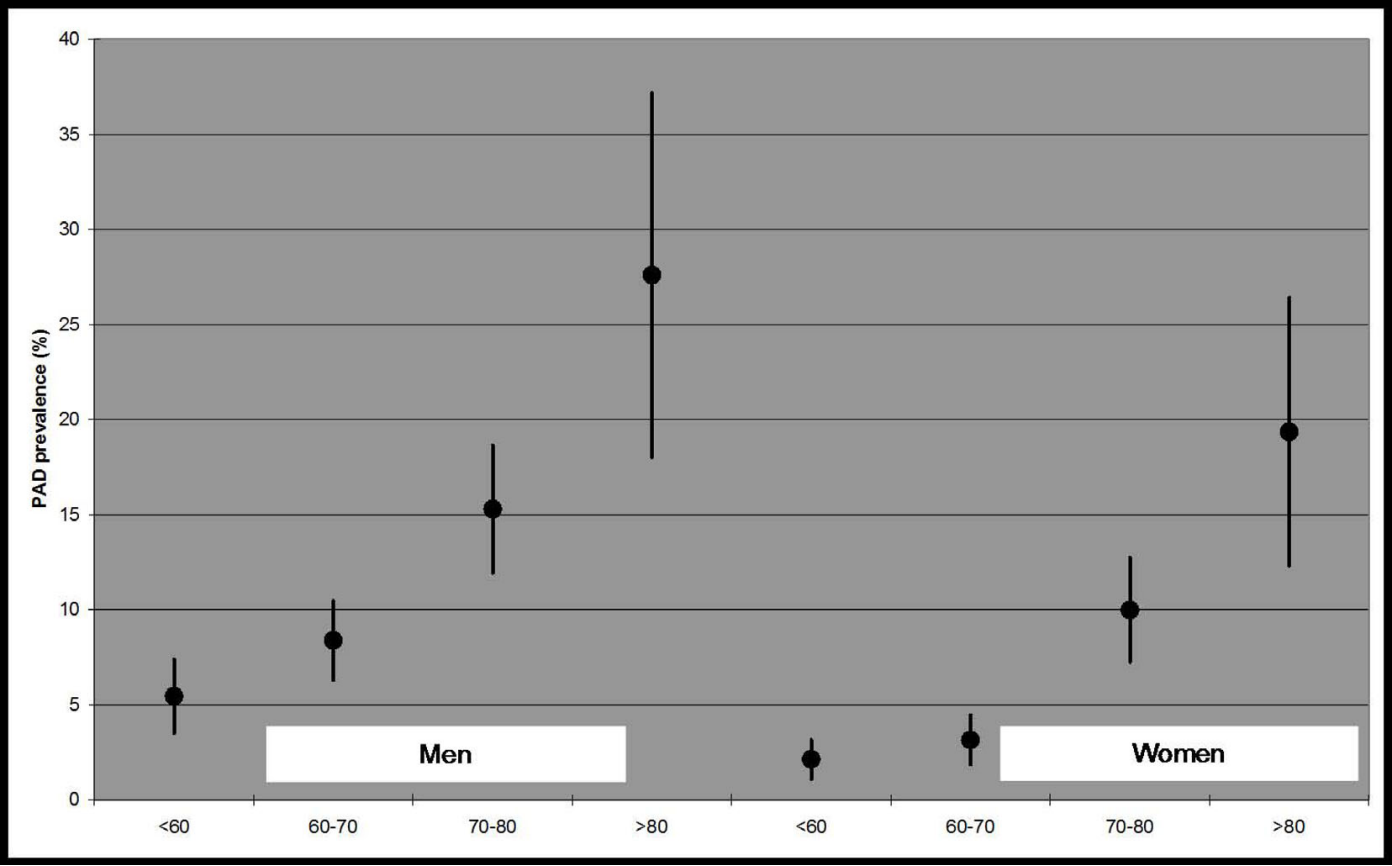
**Prevalence of peripheral arterial disease and 95% confidence interval by age and sex**.

The percentage of participation in the study was of 63%, with 3786 participants >49 years [1746 men (46.1%) and 2040 women (53.9%)] with a mean age of 64.9 years ± 8.9 being included. Of these, 286 patients had an ankle-arm index < 0.9. The prevalence of peripheral arterial disease in the total population was 7.6% (95% confidence interval 6.7-8.4) (men 10.2% (9.2-11.2), women 5.3% (4.6-6.0); p < 0.001). For each 10 years of increased age, the prevalence of peripheral arterial disease doubled. Figure 1. Arterial calcification was diagnosed in 235 patients, representing 6.2% (5.5-7.0) of the patients (men: 8.5% (7.6-9.4), women: 4.2% (3.6-4.8); p < 0.001) who were excluded from the analysis of the factors associated with peripheral arterial disease.

Table 1 describes the sample, excluding the patients with arterial calcification according to whether peripheral arterial disease was present or not. Intermittent claudication was present in 10.0% of the subjects. Only 19.0% of the patients with peripheral arterial disease had been previously diagnosed. Of those who had not been previously diagnosed 29.3% presented symptoms of IC. More than 36.8% of the subjects presented obesity. Most of the patients were classified as having low or intermediate cardiovascular risk according to the tables, while 10.8% had already presented cardiovascular disease.

**Table 1:** Sample characteristics by peripheral arterial disease

	PAD n = 286	Control^a^ n = 3265	*p*-value^b^
Gender			< 0.001
Men	11.1 (178)	88.9 (1419)	
Women	5.5 (108)	94.5 (1846)	
Age (years) (mean ± SD)	70.70 ± 9.26	64.22 ± 8.60	< 0.001
Education			< 0.001
Illiterate	14.2 (30)	85.8 (182)	
Primary school	8.1 (196)	91.9 (2229)	
Secondary school	5.4 (27)	94.6 (477)	
High school	5.7 (7)	94.3 (116)	
University	3.4 (4)	96.6 (112)	
Occupation status			< 0.001
Currently working	3.6 (30)	96.4 (813)	
Housewife	4.7 (34)	95.3 (689)	
Retired	12.2 (192)	87.8 (1380)	
Unemployed	3.9 (6)	96.1 (148)	
Disabled	11.1 (15)	88.9 (120)	
Other	6.3 (3)	93.8 (45)	
Ability to perform physical activity			< 0.001
No limitation	3.8 (51)	96.2 (1291)	
Mild limitation	8.5 (150)	91.5 (1618)	
Only able to do light activity	19.9 (72)	80.1 (289)	
Breathless with any activity	29.3 (12)	70.7 (29)	
Walking (hours/week)			0.376
0-3	7.2 (65)	92.8 (844)	
>3-7	8.0 (61)	92.0 (697)	
>7	6.4 (86)	93.6 (1252)	
Tobacco smoking			< 0.001
Never smoker	5.3 (104)	94.7 (1871)	
Former smoker	11.3 (108)	88.7 (844)	
Current smoker	11.9 (74)	88.1 (550)	
Claudication (people with leg pain when walking and no pain when stop)			< 0.001
No	5.9 (184)	94.1 (2952)	
Yes	26.9 (94)	73.1 (255)	
Obesity			0.164
Underweight/Average (BMI<25)	9.7 (61)	90.3 (566)	
Overweight (25≤BMI<30)	7.3 (118)	92.7 (1496)	
Obese (BMI ≥ 30)	8.2 (107)	91.8 (1198)	
Waist circumference			0.035
1st tertile^c^	6.9 (78)	93.1 (1060)	
2nd tertile	7.6 (89)	92.4 (1076)	
3rd tertile	9.7 (118)	90.3 (1104)	
Hypertension (medical record)			< 0.001
No	4.7 (88)	95.3 (1791)	
Yes	11.9 (193)	88.1 (1431)	
Hypercholesterolemia (medical record)			< 0.001
No	6.0 (108)	94.0 (1691)	
Yes	10.2 (170)	89.8 (1501)	
Blood analysis (mean ± SD)			
Total cholesterol (mg/dL)	210.03 ± 42.10	216.53 ± 38.48	0.007
HDL (mg/dL)	52.30 ± 14.09	56.03 ± 14.48	< 0.001
Triglycerides (blood analysis)			< 0.001
Normal	6.9 (181)	93.1 (2447)	
High (≥ 150 mg/dL)	11.8 (105)	88.2 (786)	
Diabetes (medical record)			< 0.001
No	6.4 (191)	93.6 (2802)	
Yes	17.0 (95)	83.0 (463)	
Stroke (medical record)			< 0.001
No	7.6 (260)	92.4 (3176)	
Yes	24.4 (22)	75.6 (68)	
Transient ischaemic attack (medical record)			0.001
No	7.7 (266)	92.3 (3167)	
Yes	17.5 (14)	82.5 (66)	
Angor (medical record)			< 0.001
No	7.5 (253)	92.5 (3105)	
Yes	19.0 (31)	81.0 (132)	
Myocardial infarction (medical record)			< 0.001
No	7.2 (245)	92.8 (3148)	
Yes	29.0 (38)	71.0 (93)	
Framingham index (aged≤74)			< 0.001
<10	2.9 (32)	97.1 (1081)	
10-20	5.5 (65)	94.5 (1120)	
>20	13.0 (81)	87.0 (542)	
REGICOR index (aged≤74))			< 0.001
<5	3.1 (41)	96.9 (1295)	
5-10	7.0 (89)	93.0 (1177)	
>10	15.0 (48)	85.0 (271)	
SCORE index (aged<65)			< 0.001
<2.5	2.4 (27)	97.6 (1105)	
2.5-5	4.0 (18)	96.0 (427)	
>5	10.9 (37)	89.1 (302)	
Metabolic syndrome (NCEP)			< 0.001
No	7.2 (198)	92.8 (2559)	
Yes	11.4 (88)	88.6 (685)	

All values are presented as prevalence of healthy and peripheral arterial disease as percent (n) unless otherwise specified. SD: standard deviation. BMI: Body mass index expressed in Kg/m^2^.

^a ^Patients with calcification excluded.

^b ^p value for a chi-squared test (categorical variable) and t-test (continuous variable). Fisher exact test and rank sum tests have also been performed for categorical and continuous variables respectively leading to similar p values.

^c ^Sex-specific tertiles. Cut points are 97 cm and 105 cm for men and 90 cm and 101 cm for women.

### Risk factors for the presence of peripheral arterial disease according to logistic regression analysis

Table 2 shows the results of the individual analysis of the risk factors associated with the presence of peripheral arterial disease according to the LRM adjusted for age, sex and centre and table 3 describes the results of the multivariate analysis according to gender. Walking more than 7 hours weekly (OR 0.67) and overweight/obesity (OR 0.57) were protector factors on multivariate analysis, although on stratification by sex, they were only statistically significant in women. Male gender (OR 1.62), age (OR 2.00 per 10 years), difficulty in performing physical exercise (OR 7.08 breathlessness performing any activity) and smoking (OR 3.83 for current smokers) were positively associated with peripheral arterial disease in both sexes. Hypertension (OR 1.85), diabetes (OR 2.01) and previous CDV (OR 2.19) were associated with peripheral arterial disease overall and among men, and hypercholesterolemia (OR 1.55) and high triglycerides (OR 1.55) were associated with peripheral arterial disease overall and among women only. Variables only significant for one gender in the multivariate analysis showed a similar trend in both sexes. Although education, occupation and IC were associated with peripheral arterial disease in the age- and sex-adjusted models, these factors had no significant effect in the multivariate models. Stroke, transient ischaemic attack, angina or and myocardial infarction were related to peripheral arterial disease in the age- and sex-adjusted model but were collapsed into a single cardiovascular disease variable in the multivariate model providing better AIC indexes. Information regarding cardiovascular disease risk tables or metabolic syndrome was not included in the multivariate model since most of their components were already included. Nevertheless, a significant association (table 2) was observed between the metabolic syndrome and peripheral arterial disease [OR 1.79 CI 95% (1.36-2.36)] and these variables showed a positive and significant association with peripheral arterial disease in the age and sex adjusted models.

**Table 2:** Association between peripheral arterial disease and potential risk factors. Logistic regression models adjusted by age and sex

	OR (95%CI)	*p*-value for trend
Men	2.13 (1.65-2.75)	
Age (per 10 years)	2.25 (1.96-2.60)	
Education (reference = Illiterate)		0.011
Primary school	0.65 (0.42-1.00)	
Secondary school	0.55 (0.31-0.99)	
High school	0.48 (0.20-1.16)	
University	0.29 (0.10-0.87)	
Occupation status (reference = Currently working)		
Housewife	1.02 (0.57-1.83)	
Retired	1.14 (0.70-1.86)	
Unemployed	1.10 (0.45-2.71)	
Disabled	2.50 (1.29-4.88)	
Other	1.76 (0.51-6.05)	
Ability to perform physical activity (reference = No limitation)		< 0.001
Mild limitation	2.07 (1.47-2.92)	
Only able to do light activity	4.84 (3.19-7.36)	
Breathless with any activity	6.70 (3.10-14.45)	
Walking (hours/week) (reference = 0-3)		0.002
>3-7	0.88 (0.60-1.29)	
>7	0.58 (0.40-0.82)	
Tobacco smoking (reference = Never smoker)		< 0.001
Former smoker	2.14 (1.45-3.17)	
Current smoker	3.91 (2.58-5.93)	
Claudication	5.00 (3.73-6.71)	
Obesity (reference = BMI<25)		0.685
Overweight (25≤BMI<30)	0.67 (0.48-0.94)	
Obese (BMI ≥30)	0.85 (0.60-1.20)	
Waist circumference		0.174
2nd tertile^a^	1.02 (0.73-1.41)	
3rd tertile	1.23 (0.90-1.67)	
Hypertension	1.99 (1.51-2.62)	
Hypercholesterolemia	1.86 (1.43-2.41)	
Triglycerides ≥ 150 mg/dL	2.05 (1.58-2.66)	
Diabetes	2.39 (1.82-3.15)	
Stroke	2.92 (1.74-4.90)	
Transient ischaemic attack	1.73 (0.93-3.20)	
Angor	1.90 (1.23-2.93)	
Myocardial infarction	3.81 (2.50-5.82)	
Framingham index (aged≤74)		< 0.001
10-20	1.42 (0.88-2.29)	
>20	2.57 (1.49-4.43)	
REGICOR index (aged≤74)		< 0.001
5-10	1.60 (1.05-2.43)	
>10	2.61 (1.55-4.38)	
SCORE index (aged<65)		< 0.001
2.5-5	1.43 (0.71-2.89)	
>5	3.99 (2.00-7.95)	
Metabolic syndrome (NCEP)	1.79 (1.36-2.36)	

Patients with calcification excluded.

All models adjusted for age, sex and centre.

BMI: Body mass index.

^a^Sex-specific tertiles. Cut points are 97 cm and 105 cm for men and 90 cm and 101 cm for women.

**Table 3:** Association between peripheral arterial disease and potential risk factors. Multivariate logistic regression models by sex.

	Total	Men	Women
	**OR (95%CI)**	**OR (95%CI)**	**OR (95%CI)**

Men	1.62 (1.01-2.59)		
Age (×10 years)	2.00 (1.64-2.44)	1.83 (1.42-2.36)	2.17 (1.52-3.09)
PA Mild limitation^a^	1.77 (1.17-2.68)	2.16 (1.33-3.51)	0.92 (0.42-2.03)
PA Only able light activity^a^	3.64 (2.16-6.13)	4.09 (2.12-7.91)	2.39 (0.97-5.90)
PA Breathless any activity^a^	7.08 (2.61-19.2)	7.47 (1.87-29.8)	5.40 (1.14-25.7)
>7 h/w walking^b^	0.67 (0.49-0.94)	0.82 (0.55-1.23)	0.43 (0.22-0.82)
Former smoker^c^	2.19 (1.34-3.58)	2.48 (1.26-4.90)	1.47 (0.52-4.11)
Current smoker^c^	3.83 (2.23-6.58)	4.18 (2.01-8.69)	4.00 (1.59-10.1)
BMI ≥25 Kg/m^2^	0.57 (0.38-0.87)	0.65 (0.39-1.08)	0.43 (0.20-0.92)
Hypertension	1.85 (1.29-2.65)	1.78 (1.16-2.72)	2.03 (0.99-4.149
Hypercholesterolemia	1.55 (1.11-2.18)	1.36 (0.90-2.07)	1.87 (1.03-3.40)
High triglycerides	1.55 (1.10-2.19)	1.27 (0.82-1.98)	2.29 (1.29-4.05)
Diabetes	2.01 (1.42-2.83)	2.27 (1.50-3.44)	1.59 (0.85-2.99)
Cardiovascular disease	2.19 (1.52-3.15)	2.54 (1.66-3.90)	1.43 (0.67-3.02)

Patients with calcification excluded.

All models adjusted for the variables included in the table and centre.

PA: Physical activity (reference = No limitation), BMI: Body mass index

^a ^p for trend are < 0.001 (total and men) and 0.009 (women) for physical activity.

^b^Reference = less than or equal to 7 hours walking per week

^c ^p for trend are < 0.001 (total and men) and 0.003 (women) for smoking.

## Discussion

The results of the present study indicate that the prevalence of peripheral arterial disease using the ankle-arm index (7.6%) is lower in a country of low cardiovascular risk such as Spain in relation to other countries with a higher risk [8-13]. Thus, our results coincide with studies which have reported lower values of cardiovascular disease such as ischaemic heart disease [25]. In another Spanish study [15], the prevalence of peripheral arterial disease was lower (4.5%). Nonetheless, the age of the subjects studied was from 35 to 79 years which could explain the difference with our results considering that it is well known that the prevalence of cardiovascular disease increases with age [7].

The prevalence of peripheral arterial disease in the general population is not well known in countries of low risk. In Spain, all the previous studies have been undertaken in populations at high risk or well selected for different factors: male sex, smokers, diabetics, metabolic syndrome or previous cardiovascular disease. In these studies the prevalence ranged from 3.9% to 26.2% according to the population studied [25-30], with the high prevalence among hospitalized (26.2%) [27] and diabetic patients (21.4%) [30] being of note. The low prevalence of peripheral arterial disease in countries with low cardiovascular risk [25] but a high prevalence of cardiovascular risk factors (French paradox) could be explained by protective factors in Mediterranean countries, such as dietetic factors. Studies in Spain have confirmed this low prevalence of peripheral arterial disease [15,16], coronary heart disease [31,32] and stroke [33,34] compared with studies conducted in Northern Europe.

One alarming data of our study was the under diagnosis of peripheral arterial disease of 81.0% which, according to some authors, may be attributed to the large number of patients (80-90%) who remain asymptomatic and that the ankle arm index is not routinely evaluated in primary care offices. It is important to note that in the present study 33.8% of the patients with a pathologic ankle arm index presented clinical manifestations of IC, as defined by the Edinburgh questionnaire [8,35,36]. The patients may attribute this symptomatology to other diseases or pathological processes. In most of the studies published, the IC values among patients with peripheral arterial disease were between 5% and 29.6% [10,12], with the exception of the study by Coni et al. [37] in which the prevalence of IC was very high (37.5%) and similar to that of our study, although, in contrast with the previous study, we used the Edinburgh questionnaire.

The proportion of patients with arterial calcification was of note (6.2%). Its clinical significance has been little studied. Nonetheless, it has recently been shown to be associated with a greater risk of morbi-mortality [38] although not as important as an ankle arm index < 0.9. These patients were excluded from the analysis of the associated factors to allow better comparability among healthy patients and those with peripheral arterial disease. This will be studied in the future.

Similar to other studies, the present series found a higher prevalence of peripheral arterial disease in men which progressively rose with age in both sexes. In women, a protector effect was found with physical exercise (walking >7 hours per week) and overweight, although this was not statistically significant in men. This protector effect of physical exercise may be justified by a greater development of collateral circulation, which is currently one of the therapeutic recommendations in these patients. The protector effect of obesity and overweight independent of physical exercise and other risk factors may be surprising, but a better prognosis has also been described in patients with obesity in heath failure and a lower incidence of acute myocardial infarction [39,40]. However, we did not find any relationship with waist circumference, although on adjusting for the body mass index (BMI) we did find a relationship with the second [OR 1.25 (0.87-1.81)] and the third tertile [OR 1.56 (1.02-2.37)] compared to the first tertile, which disappeared on adjustment for the remaining variables in the multivariate model.

With regard to studies evaluating the association with different factors, our results coincide in age [8-13], male gender [8-13], hypertension [9-11], diabetes [11,12], smoking [10-12], and history of cardiovascular disease [9,10]. In the present study we found an association with hypercholesterolemia and hypertriglyceridemia in women. In other studies [11,12], an association has also been reported with hypercholesterolemia, while the results related to triglycerides in the literature are contradictory [9].

With respect to limitations of the study, as usually occurs in population-based studies in Spain, women were slightly over-represented [14]. Although the data from the census were not used, in Spain the use of the population assigned to the primary care centres is preferable since it is more exhaustive and updated than that of the census [41]. On the other hand, the cross-sectional design of the study did not allow determination of the causal effect of the different factors associated with peripheral arterial disease.

The technical ease of the ankle-arm index and it ready adoption in the primary care offices has replaced the use of carotid echo-Doppler for detecting patients with high cardiovascular risk. However, a large proportion of the population has an intermediate risk, thus it remains to be defined in which patients ankle arm index should be a priority. According to the present study these patients would include: males, aged over 60 years, smokers or ex-smokers, those with clinical manifestations of IC or difficulty in performing physical activity, patients with hypertension, diabetes or hypertriglyceridemia. The addition of ankle-arm index in prediction tables of cardiovascular risk can improve the sensitivity and positive predictive values, especially in patients with intermediate or low cardiovascular risk.

## Conclusions

In summary, the prevalence of peripheral arterial disease in our population of low-medium risk is lower compared to studies carried out in countries of high risk. Our results coincide with most of the studies published on risk factors for peripheral arterial disease, but the protector effect of physical exercise and overweight is of note. It is necessary to determine the prognostic value of peripheral arterial disease compared to cardiovascular disease in a longitudinal study and to define the population in whom the use of the ankle arm index should be a priority to thereby correctly stratify the cardiovascular risk of our population beyond the use of the tables for risk of cardiovascular disease. Further studies are required to clarify the clinical significance and the association with cardiovascular risk of patients with arterial calcification and those in whom the ankle arm index cannot be determined.

## Abbreviations

(CVD): Cardiovascular disease; (PAD): Peripheral arterial disease; (AAI): Ankle-arm index; (IC): Intermittent claudication; (LRM): Logistic regression models; (AIC): Akaike Information Criteria; (BMI): Body mass index.

## Competing interests

The authors declare that they have no competing interests.

## Authors' contributions

MTA, JMB, MS, RF, PT, MV, MDR, JL and MB participated in the design of the study; MTA, JMB, MS, RF, PT, CA, JL and AS contributed to the coordination study; GP participated in the statistical calculations. All the authors have read and approved the final manuscript.

## Pre-publication history

The pre-publication history for this paper can be accessed here:

http://www.biomedcentral.com/1471-2458/10/38/prepub

## References

[B1] Eurostat, Atlas de Mortalidad: mortalidad cardiovascular http://ec.europa.eu/health/ph_information/dissemination/diseases/cardiovascular_es.print.htm

[B2] Kiechl S Willeit J The natural course of atherosclerosis. Part I: incidence and progression Arterioscler Thromb Vasc Biol 1999 19 1484 1490 10364079 10.1161/01.atv.19.6.1484

[B3] Grundy SM Cleeman JI Daniels SR Donato KA Eckel RH Franklin BA Gordon DJ Krauss RM Savage PJ Smith SC Jr Spertus JA Costa F Diagnosis and management of the metabolic syndrome: an American Heart Association/National Heart, Lung, and Blood Institute Scientific Statement Circulation 2005 112 2735 52 10.1161/CIRCULATIONAHA.105.169404 16157765

[B4] Grau M Marrugat J Risk functions and the primary prevention of cardiovascular disease Rev Esp Cardiol 2008 61 404 06 18405521

[B5] Newman AB Shemanski L Manolio TA Cushman M Mittelmark M Polak JF Powe NR Siscovick D Ankle-arm index as a predictor of cardiovascular disease and mortality in the Cardiovascular Health Study. The Cardiovascular Health Study Group Arterioscler Thromb Vasc Biol 1999 19 538 45 10073955 10.1161/01.atv.19.3.538

[B6] Criqui MH Langer RD Fronek A Feigelson HS Klauber MR McCann TJ Browner D Mortality over a period of 10 years in patients with peripheral arterial disease N Engl J Med 1992 326 381 386 1729621 10.1056/NEJM199202063260605

[B7] Ankle Brachial Index Collaboration Fowkes FG Murray GD Butcher I Heald CL Lee RJ Chambless LE Folsom AR Hirsch AT Dramaix M deBacker G Wautrecht JC Kornitzer M Newman AB Cushman M Sutton-Tyrrell K Fowkes FG Lee AJ Price JF d'Agostino RB Murabito JM Norman PE Jamrozik K Curb JD Masaki KH Rodríguez BL Dekker JM Bouter LM Heine RJ Nijpels G Stehouwer CD Ferrucci L McDermott MM Stoffers HE Hooi JD Knottnerus JA Ogren M Hedblad B Witteman JC Breteler MM Hunink MG Hofman A Criqui MH Langer RD Fronek A Hiatt WR Hamman R Resnick HE Guralnik J McDermott MM Ankle brachial index combined with Framingham Risk Score to predict cardiovascular events and mortality: a meta-analysis JAMA 2008 300 197 208 10.1001/jama.300.2.197 18612117 PMC2932628

[B8] Fowkes FG Housley E Cawood EH Macintyre CC Ruckley CV Prescott RJ Edinburgh Artery Study: Prevalence of asymptomatic and symptomatic peripheral arterial disease in the general population Int J Epidemiol 1991 20 384 92 10.1093/ije/20.2.384 1917239

[B9] Murabito JM Evans JC Nieto K Larson MG Levy D Wilson PW Prevalence and clinical correlates of peripheral arterial disease in the Framingham Offspring Study Am Heart J 2002 143 961 5 10.1067/mhj.2002.122871 12075249

[B10] Meijer Wouter T Hoes Arno W Rutgers Dominique Bots Michiel L Hofman Albert Grobbee Diederich E Peripheral arterial disease in the elderly: The Rotterdam study Arterioscler Thromb Vas Biol 1998 18 185 192 10.1161/01.atv.18.2.185 9484982

[B11] Stoffers HE Rinkens PE Kester AD Kaiser V Knottnerus JA The prevalence of asymptomatic and unrecognized peripheral arterial occlusive disease Int J Epidemiol 1996 25 282 90 10.1093/ije/25.2.282 9119553

[B12] Ostchega Y Paulose-Ram R Dillon CF Gu Q Hughes JP Prevalence of peripheral arterial disease and risk factors in persons aged 60 and older: data from the National Health and Nutrition Examination Survey 1999-2004 J Am Geriatr Soc 2007 55 583 9 10.1111/j.1532-5415.2007.01123.x 17397438

[B13] Weatherley BD Nelson JJ Heiss G Chambless LE Sharrett AR Nieto FJ Folsom AR Rosamond WD The association of the ankle-brachial index with incident coronary heart disease: the Atherosclerosis Risk In Communities (ARIC) study, 1987-2001 BMC Cardiovasculars Disorders 2007 7 3 10.1186/1471-2261-7-3 PMC1784111 17227586

[B14] Baena Diez JM del Val Garcia JL Tomas Pelegrina J Martinez Martinez JL Martin Penacoba R González Tejon I Epidemiología de las enfermedades cardiovasculares y factores de riesgo en atención primaria Rev Esp Cardiol 2005 58 367 73 10.1157/13073893 15847733

[B15] Ramos R Quesada M Solanas P Subirana I Sala J Vila J Masiá R Cerezo C Elosua R Grau M Cordón F Juvinyà D Fitó M Isabel Covas M Clarà A Angel Muñoz M Marrugat J on behalf of the REGICOR Investigators Prevalence of Symptomatic and Asymptomatic Peripheral Arterial Disease and the Value of the Ankle-brachial Index to Stratify Cardiovascular Risk Eur J Vasc Endovasc Surg 2009 38 305 11 10.1016/j.ejvs.2009.04.013 19515589

[B16] Blanes JI Cairols MA Marrugat J Prevalence of peripheral artery disease and its associated risk factors in Spain: The ESTIME Study Int Angiol 2009 28 20 5 19190551

[B17] Bendermacher BL Teijink JA Willigendael EM Bartelink ML Büller HR Peters RJ Boiten J Langenberg M Prins MH Symptomatic peripheral arterial disease: the value of a validated questionnaire and a clinical decision rule Br J Gen Pract 2006 56 932 7 17132381 PMC1934053

[B18] Kaiser V Kester AD Stoffers HE Kitslaar PJ Knottnerus JA The influence of experience on the reproducibility of the ankle-brachial systolic pressure ratio in peripheral arterial occlusive disease Eur J Vasc Endovasc Surg 1999 18 25 29 10.1053/ejvs.1999.0843 10388635

[B19] Stoffers HE Kester AD Kaiser V Rinkens PE Kitslaar PJ Knottnerus JA The diagnostic value of the measurement of the ankle-brachial systolic pressure index in primary health care J Clin Epidemiol 1996 49 1401 5 10.1016/S0895-4356(96)00275-2 8970490

[B20] Guo X Li J Pang W Zhao M Luo Y Sun Y Hu D Sensitivity and specificity of ankle-brachial index for detecting angiographic stenosis of peripheral arteries Circ J 2008 72 605 10 10.1253/circj.72.605 18362433

[B21] Holland-Letz T Endres HG Biedermann S Mahn M Kunert J Groh S Pittrow D von Bilderling P Sternitzky R Diehm C Reproducibility and reliability of the ankle-brachial index as assessed by vascular experts, family physicians and nurses Vasc Med 2007 12 105 12 10.1177/1358863X07077281 17615798

[B22] Alzamora MT Baena-Díez JM Sorribes M Forés R Toran P Vicheto M Pera G Reina MD Albaladejo C Llussà J Bundó M Sancho A Heras A Rubiés J Arenillas JF PERART study Peripheral Arterial Disease study (PERART): prevalence and predictive values of asymptomatic peripheral arterial occlusive disease related to cardiovascular morbidity and mortality BMC Public Health 2007 7 348 10.1186/1471-2458-7-348 18070367 PMC2241612

[B23] Executive summary of the third report of the Nacional Colesterol Education (NCEP) Expert Panel on Detection, Evaluation and Treatment of High Cholesterol in Adults(Adults Treatment Panel III) JAMA 2001 285 2486 2497 10.1001/jama.285.19.2486 11368702

[B24] Burnham KP Anderson DR Model selection and multimodel inference: a practical information-theoretic approach 2002 New-York: Springer-Verlag 496

[B25] Tunstall-Pedoe H Kuulasmaa K Mahönen M Tolonen H Ruokokoski E Philippe Amouyel for the WHO MONICA Project Contribution of trends in survival and coronary-event rates to changes in coronary heart disease mortality: 10-year results from 37 WHO MONICA Project populations Lancet 1999 353 1547 57 10.1016/S0140-6736(99)04021-0 10334252

[B26] Planas A Clara A Marrugat J Pou JM Gasol A de Moner A Age at onset of smoking is an independent risk factor in peripheral artery disease development J Vasc Surg 2002 35 506 9 10.1067/mva.2002.120030 11877699

[B27] Manzano L Mostaza JM Suárez C Cairols M Redondo R Valdivielso P Monte R Blázquez JC Ferreira EM Trouillhet I González-Igual JJ Sánchez-Zamorano MA en Representación del Estudio MERITO Value of the ankle-brachial index in cardiovascular risk stratification of patients without known atherothrombotic disease. MERITO study Med Clin (Barc) 2007 128 241 6 10.1157/13099239 17335735

[B28] Vicente I Lahoz C Taboada M García A San Martín MA Terol I Laguna F García-Iglesias F Mostaza JM Prevalence of an abnormal ankle-brachial index in relation to the cardiovascular risk estimated by the Framingham function Med Clin (Barc) 2005 124 641 4 10.1157/13074738 15882509

[B29] Lahoz C Vicente I Laguna F García-Iglesias MF Taboada M Mostaza JM Metabolic syndrome and asymptomatic peripheral artery disease in subjects over 60 years of age Diabetes Care 2006 29 148 50 10.2337/diacare.29.01.06.dc05-1617 16373915

[B30] Bundo M Auba J Valles R Torner O Perez AM Massons J Peripheral arterial disease in Diabetes mellitus Aten Primaria 1998 22 5 11 9741155

[B31] Masia R Pena A Marrugat J Sala J Vila J Pavesi M Covas M Aubo C Elosua R High prevalence of cardiovascular risk factors in Gerona, Spain, a province with low myocardial infarction incidence. REGICOR Investigators J Epidemiol Community Health 1998 52 707 715 10.1136/jech.52.11.707 10396503 PMC1756647

[B32] Tunstall-Pedoe H Kuulasmaa K Mahönen M Tolonen H Ruokokoski E Philippe Amouyel for the WHO MONICA Project Contribution of trends in survival and coronary-event rates to changes in coronary heart disease mortality: 10-year results from 37 WHO MONICA Project populations Lancet 1999 353 1547 57 10.1016/S0140-6736(99)04021-0 10334252

[B33] Boix R del Barrio JL Saz P Reñé R Manubens JM Lobo A Gascón J de Arce A Díaz-Guzmán J Bergareche A Bermejo-Pareja F de Pedro-Cuesta J Spanish Epidemiological Study Group on Ageing Stroke prevalence among the Spanish elderly: an analysis based on screening surveys BMC Neurol 2006 6 36 10.1186/1471-2377-6-36 17042941 PMC1626484

[B34] Rothwell PM Coull AJ Giles MF Howard SC Silver LE Bull LM Gutnikov SA Edwards P Mant D Sackley CM Farmer A Sandercock PA Dennis MS Warlow CP Bamford JM Anslow P Oxford Vascular Study Change in stroke incidence, mortality, case-fatality, severity, and risk factors in Oxfordshire, UK from 1981 to 2004 (Oxford Vascular Study) Lancet 2004 363 1925 1933 10.1016/S0140-6736(04)16405-2 15194251

[B35] Meijer WT Hoes AW Rutgers D Bots ML Hofman A Grobbee DE Peripheral arterial disease in the elderly: The Rotterdam study Arterioscler Thromb Vasc Biol 1998 18 185 92 9484982 10.1161/01.atv.18.2.185

[B36] Fowkes FG Housley E Cawood EH Macintyre CC Ruckley CV Prescott RJ Edinburgh Artery Study: Prevalence of asymptomatic and symptomatic peripheral arterial disease in the general population Int J Epidemiol 1991 20 384 92 10.1093/ije/20.2.384 1917239

[B37] Coni M Tennison B Troup M Prevalence of lower extremity arterial disease among elderly people in the community Br J Gen Pract 1992 42 149 152 1586550 PMC1371892

[B38] Fowkes FG Murray GD Butcher I Heald CL Lee RJ Chambless LE Folsom AR Hirsch AT Dramaix M deBacker G Wautrecht JC Kornitzer M Newman AB Cushman M Sutton-Tyrrell K Fowkes FG Lee AJ Price JF d'Agostino RB Murabito JM Norman PE Jamrozik K Curb JD Masaki KH Rodríguez BL Dekker JM Bouter LM Heine RJ Nijpels G Stehouwer CD Ferrucci L McDermott MM Stoffers HE Hooi JD Knottnerus JA Ogren M Hedblad B Witteman JC Breteler MM Hunink MG Hofman A Criqui MH Langer RD Fronek A Hiatt WR Hamman R Resnick HE Guralnik J McDermott MM Ankle brachial index combined with Framingham Risk Score to predict cardiovascular events and mortality: a meta-analysis. Ankle Brachial Index Collaboration JAMA 2008 300 197 208 10.1001/jama.300.2.197 18612117 PMC2932628

[B39] Kenchaiah S Gaziano JM Vasan RS Impact of obesity on the risk of heart failure and survival after the onset of heart failure Med Clin North Am 2004 88 1273 94 10.1016/j.mcna.2004.04.011 15331317

[B40] Nicoletti I Cicoira M Morando G Benazzi C Prati D Morani G Rossi A Zardini P Vassanelli C Impact of body mass index on short-term outcome after acute myocardial infarction: does excess body weight have a paradoxical protective role? Int J Cardiol 2006 107 395 9 10.1016/j.ijcard.2005.04.005 16503262

[B41] Taxes d'Incidència i prevalença a l'Atenció Primària Mètodes per a la seva obtenció 2002 Barcelona: Fundació Jordi Gol i Gurina

